# Supplementary material for: Cytotoxicity of Biodegradable Zinc and Its Alloys: A Systematic Review
Source: J Funct Biomater. 2023 Apr 7;14(4):206. doi: 10.3390/jfb14040206 (PMC10144193; doi:10.3390/jfb14040206)
Supplement: Supplementary file 1 [file jfb-14-00206-s001.zip › Table S1.Electronic database and search strategy.pdf]

Table S1. Electronic database and search strategy. (28/09/2022)

**PubMed**		
#1 (zinc[MeSH Terms] OR Zn[Title/Abstract] OR Zn alloy[Title/Abstract] OR Zn-based alloy[Title/Abstract])	#2 (biodegradable metal[Title/Abstract] OR degradable metal[Title/Abstract] OR biodegradable[Title/Abstract] OR degradable[Title/Abstract] OR absorbable[Title/Abstract] OR biodegradable implant*[Title/Abstract] OR biodegradable fixation[Title/Abstract] OR absorbable implant*[Title/Abstract] OR bioabsorbable implant*[Title/Abstract] OR biodegrading implant*[Title/Abstract])	#3 (toxicity tests[MeSH Terms] OR cytotoxicity[Title/Abstract] OR cytocompatibility[Title/Abstract] OR toxicity test*[Title/Abstract] OR mutagenicity tests[MeSH Terms] OR genotoxicity test*[Title/Abstract] OR genotoxic effect*[Title/Abstract] OR genotoxicity[Title/Abstract] OR biocompatible materials[MeSH Terms] OR biomaterial*[Title/Abstract] OR cell culture techniques[MeSH Terms] OR cell culture*[Title/Abstract] OR cell survival[MeSH Terms] OR cell viabilit*[Title/Abstract])
#1 AND #2 AND #3		
**Scopus**		
#1 (TITLE-ABS-KEY ((zinc OR Zn OR “Zn alloy” OR “Zn-based alloy”)))	#2 (TITLE-ABS-KEY (("biodegradable metal" OR "degradable metal" OR biodegradable OR degradable OR absorbable OR "biodegradable implant*" OR "biodegradable fixation" OR" absorbable implant*"OR "bioabsorbable implant*" OR "biodegrading implant*")))	#3 (TITLE-ABS-KEY ((cytotoxicity OR cytocompatibility OR "toxicity test" OR "genotoxicity test" OR "genotoxic effect" OR genotoxicity OR biomaterial OR "cell culture" OR "cell viability")))
#1 AND #2 AND #3		
**Web Of Science**		
#1 TS= (zinc OR Zn OR “Zn alloy” OR “Zn-based alloy”)	#2 TS= ("biodegradable metal" OR "degradable metal" OR biodegradable OR degradable OR absorbable OR "biodegradable implant*" OR "biodegradable fixation" OR "absorbable implant*" OR "bioabsorbable implant*" OR "biodegrading implant*")	#3 TS= (cytotoxicity OR cytocompatibility OR “toxicity test” OR “genotoxicity test” OR “genotoxic effect” OR genotoxicity OR “biomaterial” OR “cell culture” OR “cell viability”)
#1 AND #2 AND #3		
